# Baseline platelet parameters for predicting early platelet response and clinical outcomes in patients with non-cardioembolic ischemic stroke treated with clopidogrel

**DOI:** 10.18632/oncotarget.21622

**Published:** 2017-10-07

**Authors:** Wenxian Li, Xiaomei Xie, Di Wei, Shijun Zhang, Yuanling Wu, Xuejun Fu, Zhen Jing, Weibiao Lu, Xinqiang Lai, Li’an Huang

**Affiliations:** ^1^ Department of Neurology, The First Affiliated Hospital, Jinan University, Guangzhou, Guangdong, 510632, P.R. China; ^2^ Department of Neurology, People’s Hospital of Zengcheng District, Guangzhou, Guangdong, 510180, P.R. China; ^3^ Department of Neurology, People’s Hospital, Second Clinical College, Jinan University, Shenzhen, 518020, P.R. China; ^4^ Analysis and Testing Center, Jinan University, Guangzhou, Guangdong, 510632, P.R. China; ^5^ Department of Urology, Xijing Hospital, Fourth Military Medical University, Xi'an, Shanxi, 710032, P.R. China

**Keywords:** mean platelet volume, platelet count, clopidogrel resistance, non-cardioembolic ischemic stroke, platelet response

## Abstract

**Purpose:**

The present study investigated whether routine baseline platelet parameters(BPPs) detected before clopidogrel therapy in acute non-cardioembolic ischemic stroke(NCIS) could predict early platelet response and future clinical outcomes.

**Results:**

The *CYP2C19* polymorphisms constituted independent risk factors for LCR. The number of female patients, the incidence of diabetes mellitus (DM), the level of low-density lipoprotein(LDL) cholesterol, and the neutrophil-to-lymphocyte ratio(NLR) were significantly high in the clinical clopidogrel resistance (CCR) group. However, none of the BPPs had a significant association with laboratory clopidogrel resistance (LCR) or discriminated with the cut-off values regarding LCR or CCR. The patients were divided into two groups according to the average mean platelet volume(MPV) or platelet count(PC). We found that the HbA1c level, the number of female patients, and the CCR were higher in the groups with elevated MPV (≥ 10.6fL) and PC (≥ 235 × 10^9^/L); the LCR, the NIHSS score at discharge, and elevated MPV and PC were risk predictors for CCR.

**Materials and Methods:**

This study included 196 patients with acute NCIS who underwent routine blood tests upon admission, were treated with clopidogrel, and were followed up for 6 months. Early platelet response was assessed and the *CYP2C19* genetic variants were screened for. All participants were categorized into either laboratory clopidogrel resistance(LCR) or clinical clopidogrel resistance (CCR) groups.

**Conclusions:**

Elevated baseline MPV and PC before clopidogrel therapy, as well as CYP2C19 gene variants, should be included in a risk algorithm for NCIS. Furthermore, other nongenetic clinical risk factors should be assessed for optimal prediction of the risk for thrombotic events because of individual variability in platelet response to clopidogrel.

## INTRODUCTION

Platelets exert a central role in the development of ischemic stroke (IS) by the pathophysiology of thrombosis. The efficacy and safety of clopidogrel in preventing subsequent thrombotic events in non-cardioembolic ischemic stroke (NCIS) are well-established [1]. However, a considerable proportion of patients (prevalence 4–30%) continue to experience recurrent ischemic thrombotic complications [2, 3]. The platelet aggregation function cannot be inhibited by clopidogrel effectively. This phenomenon might be attributed to the individual variability in the platelet response to clopidogrel therapy and may be associated with a high incidence of recurrent thrombotic events or poor prognosis, arising because of clopidogrel resistance (CR). Currently, CR is further characterized by tests on platelet functions [3] and by the analysis of genetic polymorphisms [4]. The baseline platelet parameters (BPPs) can be obtained easily and quickly upon admission. Several studies have focused on BPPs within the normal range that have been related to risk factors for cardiovascular or cerebrovascular diseases and impaired antiplatelet therapy responses [5–12]. These included an elevated mean platelet volume (MPV) [11], a high platelet–large cell ratio (P-LCR) [6, 7, 9, 13], an increased platelet distribution width (PDW) [9], an elevated platelet count (PC) [14], and high plateletcrit (PCT) [15]. However, these theories remain controversial [10, 16]. Thus, the pre-procedural predictors of the response to platelet inhibitors or CR from the perspective of cost-effectiveness, time-saving, and technical simplicity are in demand. The present study was designed to assess whether the routine baseline platelet indicators detected before clopidogrel therapy in acute NCIS can predict early platelet response and future clinical outcomes.

## RESULTS

### Relevant demographic characteristics of the patients

A total of 258 patients with NCIS fulfilled the inclusion and exclusion criteria during the study period. In total, 62 cases were excluded for the following reasons: seven patients were diagnosed with cardioembolic stroke, three patients had an undetermined etiology, 23 patients received other antiplatelet drugs after discharge, 21 did not undergo genetic analysis, and eight were lost to follow-up. Finally, 196 patients were available for further analysis. A flow chart of the current study is shown in Supplementary Figure 1. A total of 78 (39.80%) patients had laboratory clopidogrel resistance (LCR) and 37 (18.88%) patients had clinical clopidogrel resistance (CCR). Moreover, 14 patients had stroke progression during hospitalization, 17 patients developed recurrent strokes, and six patients had other ischemic vascular events during the 6-month follow-up period. The relevant demographic characteristics of the LCR or CCR groups are summarized in Table 1A and 1B.

**Table 1A:** Relevant demographic characteristics of the LCR and the non-LCR group

Characteristics	LCR (*n =* 78)	non-LCR (*n =* 118)	*P* value
***Non-genetic risk factors***			
***VRFs***			
Age (years)	63.67 ± 10.61	62.65 ± 11.60	0.280
Men, *n* (%)	55 (70.51)	81 (68.64)	0.781
Smoking history, *n* (%)	30 (38.46)	51 (43.22)	0.508
Drinking history, *n* (%)	17 (21.79)	30 (25.42)	0.560
Hypertension, *n* (%)	49 (62.82)	75(63.56)	0.916
DM, *n* (%)	26 (33.33)	25(21.19)	0.058
Dyslipidemia, *n* (%)	8 (10.26)	16 (13.56)	0.490
CHD, *n* (%)	5 (6.41)	4 (3.39)	0.323
Stroke/TIA, *n* (%)	8 (10.26)	14 (11.86)	0.727
***BPPs***			
PC (×10^9^/L)	235.73 ± 59.41	234.06 ± 62.01	0.749
MPV (fL)	10.63 ± 0.98	10.50 ± 0.89	0.317
PDW (fl)	12.86 ± 2.12	12.46 ± 1.99	0.562
PCT (%)	0.25 ± 0.06	0.24 ± 0.06	0.641
P-LCR (%)	30.19 ± 7.79	28.84 ± 7.25	0.527
***Laboratory values***			
Cholesterol (mmol/L)	4.91 ± 1.22	4.95 ± 1.75	0.758
Triglyceride (mmol/L)	1.70 ± 0.83	1.83 ± 1.40	0.106
HDL-triglyceride (mmol/L)	1.11 ± 0.26	1.17 ± 0.51	0.190
LDL-triglyceride (mmol/L)	3.13 ± 0.99	3.05 ± 0.99	0.952
HbA1C (%)	6.89 ± 2.31	6.43 ± 1.66	0.010*
NLR (%)	3.25 ± 3.08	3.01 ± 1.98	0.319
***Gene polymorphisms***			
*CYP2C19* (636G>A)			0.026*
GG	51 (65.38)	94 (79.66)	
GA/AA	27 (34.62)	24 (20.34)	
*CYP2C19* (681G>A)			0.021*
GG	38 (48.72)	77 (65.25)	
GA/AA	40 (51.28)	41 (34.75)	

**Table 1B:** Relevant demographic characteristics of the CCR and the non-CCR group

Characteristics	CCR (*n =* 37)	non-CCR (*n =* 159)	*P* value
***Non-genetic risk factors***			
***VRFs***			
Age (years)	64.35 ± 10.33	62.61 ± 11.52	0.267
Female, *n* (%)	11 (29.73)	24 (15.09)	0.036*
Smoking history, *n* (%)	14 (37.83)	67 (42.14)	0.632
Drinking history, *n* (%)	8 (21.62)	39 (24.53)	0.709
Hypertension, *n* (%)	26 (70.27)	97 (61.01)	0.294
DM, *n* (%)	15 (40.54)	34 (21.38)	0.015*
Dyslipidemia, *n* (%)	6 (16.22)	17 (10.69)	0.347
CHD, *n* (%)	3 (8.11)	6 (3.77)	0.257
Stroke/TIA, *n* (%)	5 (13.51)	17 (10.69)	0.624
***BPPs***			
PC (×10^9^/L)	253.95 ± 64.98	230.33 ± 59.45	0.514
MPV (fL)	10.36 ± 0.98	10.60 ± 0.91	0.504
PDW (fl)	12.50 ± 2.13	12.62 ± 2.05	0.886
PCT (%)	0.26 ± 0.06	0.32 ± 0.98	0.469
P-LCR (%)	28.05 ± 7.77	29.52 ± 7.75	0.830
***Laboratory values***			
Cholesterol (mmol/L)	5.15 ± 1.35	4.88 ± 1.15	0.140
Triglyceride (mmol/L)	1.84 ± 0.87	1.76 ± 1.27	0.512
HDL-triglyceride (mmol/L)	1.06 ± 0.21	1.16 ± 0.46	0.157
LDL-triglyceride (mmol/L)	3.34 ± 1.23	3.02 ± 0.92	0.006*
HbA1C (%)	6.89 ± 1.91	6.55 ± 1.96	0.722
NLR (%)	3.65 ± 4.12	2.98 ± 1.89	0.018*
***Platelet response to clopidogrel***			
LCR	22 (59.46)	55 (34.59)	0.005*
non-LCR	15 (40.54)	104 (65.41)	
***Gene polymorphisms***			
*CYP2C19* (636G>A)			0.069
GG	23 (62.16)	122 (76.73)	
GA/AA	14 (37.84)	37 (23.27)	
*CYP2C19* (681G>A)			
GG	18 (48.65)	97 (61.00)	0.169
GA/AA	19 (51.35)	62 (39.00)	

LCR, laboratory clopidogrel resistance; CCR, clinical clopidogrel resistance; VRFs, vascular risk factors; DM, diabetes mellitus; CHD, coronary heart disease; TIA, transient ischemic attack; BPPs, baseline platelet parameters; PC, platelet count; MPV, mean platelet volume; PDW, platelet distribution width; PCT, plateletocrit; P-LCR, platelet-large cell ratio; HDL, high-density lipoprotein; LDL, low-density lipoprotein; GG, wild-type gene; GA/AA, variant genotypes.

Measurement data are expressed as a mean ± standard deviation, and categorical data are expressed as *n* (%).

*P < 0.05.

The mean level of MPV was 10.6 fL. The patients were stratified into two groups according to the MPV value, namely, the low MPV group (MPV < 10.6 fL; *n* = 95) and the elevated MPV group (MPV ≥ 10.6 fL; *n* = 101). The mean level of PC was 235 × 10^9^/L. Patients were also stratified into two groups according to the PC value, namely, the low PC group (PC < 235 × 10^9^/L; *n* = 110) and the elevated PC group (PC ≥ 235 × 10^9^/L; *n* = 86). The relevant demographic characteristics of the MPV and PC groups are presented in Table 2A and 2B, respectively.

**Table 2A:** Relevant demographic characteristics of the MPV groups

Characteristics	MPV < 10.6 fL (*n =* 95)	MPV ≥ 10.6 fL (*n =* 101)	*P* value
***Non-genetic risk factors***			
*VRFs*			
Age (years)	63.06 ± 11.39	63.05 ± 11.08	0.945
Female, *n* (%)	24 (25.26)	49 (48.51)	0.001*
Smoking history, *n* (%)	44 (46.32)	37 (36.63)	0.169
Drinking history, *n* (%)	28 (29.47)	19 (18.81)	0.081
Hypertension, *n* (%)	55 (57.89)	69 (68.32)	0.130
DM, *n* (%)	27 (28.42)	24 (23.76)	0.458
Dyslipidemia, *n* (%)	13 (13.68)	11 (10.89)	0.551
CHD, *n* (%)	2 (2.11)	7 (6.93)	0.107
Stroke/TIA, *n* (%)	11 (11.58)	11 (10.89)	0.879
***BPPs***			
PC (×10^9^/L)	256.64 ± 65.21	214.23 ± 48.92	0.015*
PDW (fl)	11.15 ± 1.48	13.97 ± 1.54	0.253
PCT (%)	0.25 ± 0.06	0.36 ± 1.23	0.120
P-LCR (%)	23.38 ± 4.33	34.77 ± 6.01	0.234
***Laboratory values***			
Cholesterol (mmol/L)	4.95 ± 1.25	4.92 ± 1.13	0.251
Triglyceride (mmol/L)	1.81 ± 1.44	1.75 ± 0.94	0.384
HDL-triglyceride (mmol/L)	1.13 ± 0.26	1.15 ± 0.54	0.210
LDL-triglyceride (mmol/L)	3.08 ± 1.03	3.07 ± 0.95	0.316
HbA1C (%)	6.33 ± 1.51	6.81 ± 2.22	0.002*
NLR (%)	3.05 ± 2.07	3.16 ± 2.81	0.679
***CR***			
LCR	41 (43.16)	37 (36.63)	0.351
CCR	24 (25.26)	13 (12.87)	0.027*

**Table 2B:** Relevant demographic characteristics of the PC groups

Characteristics	PC < 235×10^9^/L (*n =* 110)	PC ≥ 235×10^9^/L (*n =* 86)	*P* value
***Non-genetic risk factors***			
***VRFs***			
Age (years)	64.15 ± 11.17	61.65 ± 11.15	0.952
Female, *n* (%)	24 (21.82)	36 (41.86)	0.003*
*Smoking history, n (%)*	49 (44.55)	32 (37.21)	0.301
Drinking history, *n* (%)	30 (27.27)	17 (19.77)	0.222
Hypertension, *n* (%)	74 (67.27)	50 (58.14)	0.188
DM, *n* (%)	28 (25.45)	23 (26.74)	0.838
Dyslipidemia, *n* (%)	10 (9.09)	14 (16.28)	0.128
CHD, *n* (%)	6 (5.45)	3 (3.49)	0.514
Stroke/TIA, *n* (%)	13 (11.82)	9 (10.47)	0.766
***BPPs***			
MPV (fL)	10.81 ± 0.94	10.23 ± 0.80	0.453
PDW (fl)	13.23 ± 2.07	11.80 ± 1.76	0.283
PCT (%)	0.32 ± 1.18	0.29 ± 0.04	0.139
P-LCR (%)	30.40 ± 7.79	26.50 ± 6.82	0.849
***Laboratory values***			
Cholesterol (mmol/L)	4.78 ± 1.12	5.13 ± 1.25	0.485
Triglyceride (mmol/L)	1.69 ± 1.40	1.89 ± 0.90	0.629
HDL-triglyceride (mmol/L)	1.13 ± 0.51	1.16 ± 0.27	0.406
LDL-triglyceride (mmol/L)	2.98 ± 0.93	3.20 ± 1.05	0.338
HbA1C (%)	6.48 ± 1.77	6.78 ± 2.15	0.044*
NLR (%)	3.35 ± 2.87	2.80 ± 1.81	0.128
***CR***			
LCR	42 (38.18)	36 (41.86)	0.602
CCR	15 (13.64)	22 (25.58)	0.034*

LCR, laboratory clopidogrel resistance; CCR, clinical clopidogrel resistance; VRFs, vascular risk factors; DM, diabetes mellitus; CHD, coronary heart disease; TIA, transient ischemic attack; BPPs, baseline platelet parameters; PC, platelet count; MPV, mean platelet volume; PDW, platelet distribution width; PCT, plateletocrit; P-LCR, platelet-large cell ratio; HDL, high-density lipoprotein; LDL, low-density lipoprotein; GG, wild-type gene; GA/AA, variant genotypes.

Measurement data are expressed as a mean ± standard deviation, and categorical data are expressed as *n* (%).

* *P* < 0.05.

### Associations of BPPs with platelet reactivity index (PRI) and gene polymorphisms

#### Associations of BPPs with PRI

**Figure 1:**
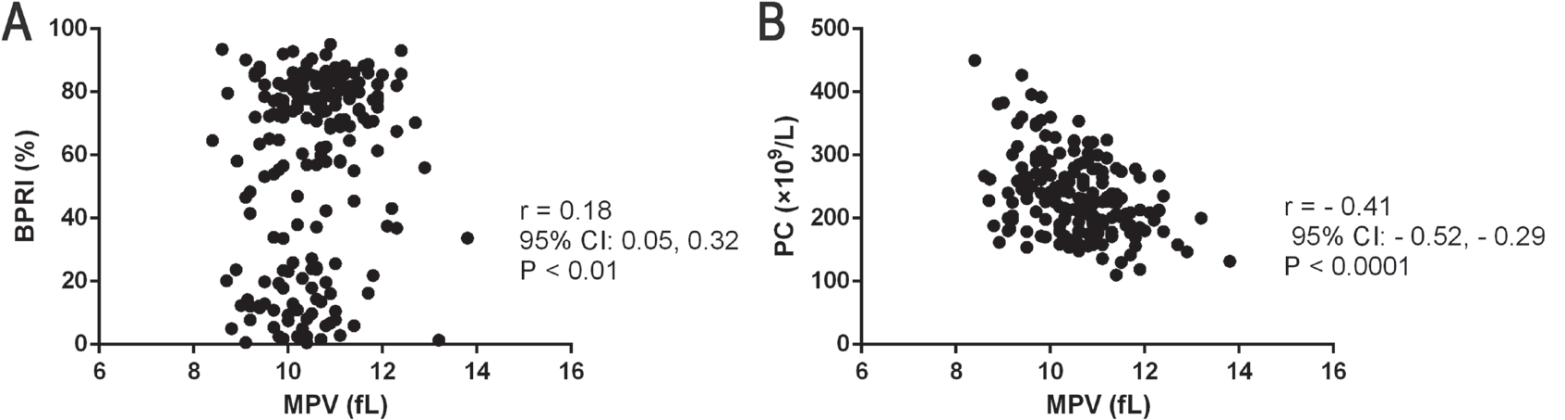
Associations between baseline BPPs and early platelet response to clopidogrel treatment for 7 days Positive correlations between: (**A**) MPV and BPRI, (**B**) MPV and PC.

A strong positive correlation was observed between the MPV and the baseline PRI (BPRI) before clopidogrel treatment of acute NCIS (r = 0.184, *P* < 0.01; Figure 1A). On the other hand, a weak, non-significant correlation was observed between MPV and the post-treatment PRI (PPRI) for 7 days; the other BPPs (PC, PDW, PCT, and P-LCR) did not correlate with BPRI or PPRI (all *P*s > 0.05).

#### Associations of BPPs with gene polymorphisms

In patients with two gene polymorphisms (*CYP2C19* [636G>A], *CYP2C19* [681G>A]), the mean values of each of the BPPs (MPV, PC, PDW, PCT, and P-LCR) were similar in the variant genotype and the wild-type gene (all *P*s > 0.05). Moreover, the variant genotypes (GG/GA) and the wild-type gene (GG) associated with each gene polymorphism did not show any significant difference between the MPV (low and elevated) and the PC groups (low and elevated; all *P*s > 0.05; Supplementary Table 1A and 1B).

### Interaction of BPPs and non-genetic risk factors

High MPV at the baseline was associated with a low PC before treatment (r = −0.413, *P* < 0.0001; Figure 1B), and the elevated MPV group had a low PC (214.23 ± 48.92 *vs*. 256.64 ± 65.21, *P* = 0.015; Table 2A).

Compared to those in the low MPV and PC groups, the number of female patients and hemoglobin A1c (HbA1c) levels were higher in the elevated MPV and PC groups (all *P*s < 0.05; Table 2A and 2B). However, no interaction effect was found between MPV/PC and the neutrophil-to-lymphocyte ratio (NLR) (all *P*s > 0.05; Table 2A and 2B).

### Associations of BPPs with LCR and CCR

#### Associations of BPPs with LCR

In this study, all BPPs (MPV, PC, PDW, PCT, and P-LCR) were similar in the LCR group as compared to the non-LCR group (all *P*s > 0.05; Table 1A).

#### Associations of BPPs with CCR

The incidence of the elevated MPV (≥ 10.6 fL) and PC (≥ 235 × 10^9^/L) was significantly higher in the CCR group than in the non-CCR group (all *P*s < 0.005). However, the other BPPs (PDW, PCT, and P-LCR) were similar (all *P*s > 0.05; Table 2A and 2B).

**Figure 2:**
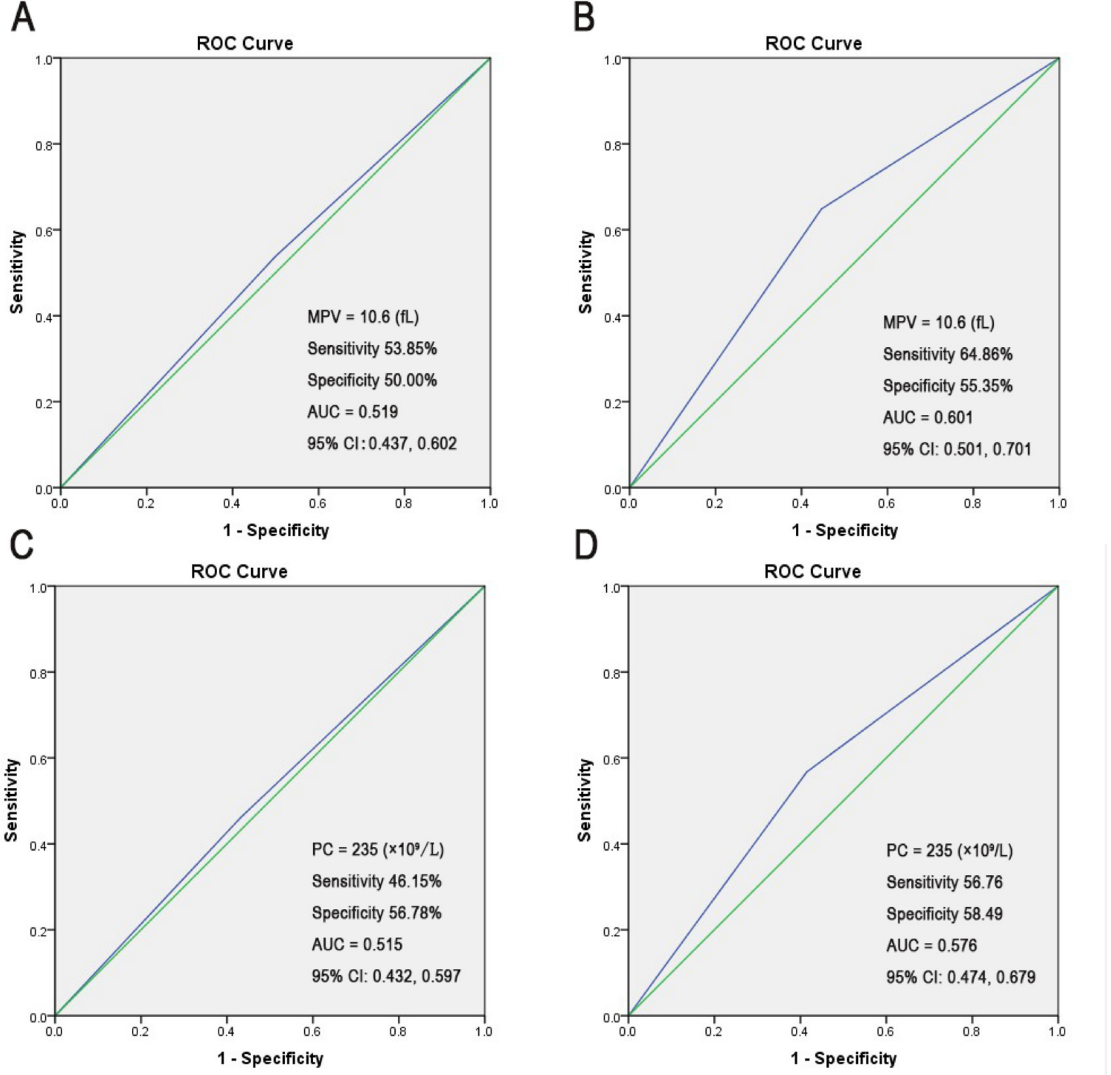
The ROC curve analysis of admission MPV/PC for predicting poor outcome (setting the cut-off value) (**A**) The MPV cut-off value was set at 10.6 fL using the ROC analysis to predict LCR. (**B**) The MPV cut-off value was set at 10.6 fL by using the ROC analysis to predict CCR. (**C**) The PC cut-off value was set at 235 × 10^9^/L by using the ROC analysis to predict LCR. (**D**) The PC cut-off value was set at 235 × 10^9^/L by using the ROC analysis to predict CCR.

In addition, receiver operating characteristic (ROC) analysis curve analysis was performed to determine the predictive cut-off value of MPV and PC in NCIS patients regarding LCR or CCR; however, no remarkable results were observed (Supplementary Figure 2). When the MPV and PC cut-off levels were set at 10.6 fL and 235 × 10^9^/L, respectively, using the ROC curve, the sensitivity and specificity were < 60% for differentiating between the groups with LCR/CCR (area under the curve [AUC] < 0.7; Figure 2).

### Risk predictors for LCR and CCR

#### Risk factors for LCR

Compared with the wild-type gene (GG), the polymorphisms of the variant genotype (GA/AA) of *CYP2C19* (636G>A) (χ^2^ = 4.972, *P* = 0.026) and *CYP2C19* (681G>A) (χ^2^ = 5.296, *P* = 0.021) were significantly associated with LCR (Table 1A). And these *CYP2C19* genotypes were risk predictors for LCR (*CYP2C19* [636G>A], odds ratio [OR] = 2.894, 95% confidence interval [CI]: 1.427–5.552, *P* = 0.003; *CYP2C19* (681G>A), OR = 2.654, 95% CI: 1.401–5.027, *P* = 0.003; Table 3A).

**Table 3A:** Non-conditional logistic regression analysis of risk predictors for LCR

	Odds ratio	95% confidence interval		*P* value
		Lower	Upper	
*CYP2C19* (636G>A)	2.894	1.427	5.552	0.003*
*CYP2C19* (681G>A)	2.654	1.401	5.027	0.003*

LCR, laboratory clopidogrel resistance.

Input variables: VRFs (age, sex, smoking history, drinking history, hypertension, DM, dyslipidemia, CHD, stroke/TIA); Gene polymorphisms [*CYP2C19* (636G>A; 681G>A)]; BPPs (PC, MPV, PDW, PCT, P-LCR); Laboratory values (Cholesterol, Triglyceride, HDL-triglyceride, LDL-triglyceride, HbA1C, NLR); Clinical scores [(NIHSS, BI, mRS, Essen) at admission and at discharge].

*P < 0.05.

The BPRI values were significantly higher in the LCR group than in the non-LCR group (70.62 ± 21.43% *vs*. 47.59 ± 31.33%, respectively; *P* < 0.001). The HbA1c level was significantly higher in the LCR group than in the non-LCR group. Nevertheless, we did not find any other non-genetic risk factors, including vascular risk factors (VRFs) (data not shown). Moreover, none of the BPPs were associated with LCR (all *P*s > 0.05; Table 1A).

In addition, the IS subtypes were not related to LCR (Supplementary Table 2A).

#### Risk factors for CCR

The CCR occurrence was significantly higher in the LCR group than in the non-LCR group (χ^2^ = 7.782, *P* = 0.005; Table 1B).

A large number of patients with CCR were female (χ^2^ = 4.383, *P* = 0.036); the CCR group had more patients with diabetes mellitus (DM) than did the non-CCR group (χ^2^ = 5.875, *P* = 0.015). In addition, the levels of low-density lipoprotein cholesterol (LDL-C) and NLR were significantly higher in patients with CCR than in the non-CCR group (CCR, F = 7.588, *P* = 0.006; non-CCR, F = 5.699, *P* = 0.018; Table 1B).

Non-conditional logistic regression analysis revealed that the risk predictors for CCR included elevated MPV (≥ 10.6 fL; OR = 2.288, 95% CI: 1.088–4.814, P = 0.029), elevated PC (≥ 235 × 10^9^/L; OR = 2.177, 95% CI: 1.050–4.512, P = 0.036), LCR (OR = 4.068, 95% CI: 1.849–8.951, *P* < 0.001), and the National Institutes of Health Stroke Scale (NIHSS) score at discharge (OR = 1.311, 95% CI: 1.087–1.581, *P* = 0.005; Table 3B).

**Table 3B:** Non-conditional logistic regression analysis of risk predictors for CCR

	Odds ratio	95% confidence interval		*P* value
		Lower	Upper	
NCIS (*n =* 196)				
MPV≥10.6	2.288	1.088	4.814	0.029*
PC≥235	2.177	1.050	4.512	0.036*
NIHSS score at discharge	1.311	1.087	1.581	0.005*
LCR	4.068	1.849	8.951	< 0.001*

Input variables: VRFs (age, sex, smoking history, drinking history, hypertension, DM, dyslipidemia, CHD, stroke/TIA); Gene polymorphisms [*CYP2C19* (636G>A; 681G>A)]; BPPs (PC, MPV, PDW, PCT, P-LCR, MPV≥10.6, PC≥235); Laboratory values (Cholesterol, Triglyceride, HDL-triglyceride, LDL-triglyceride, HbA1C, NLR); Clinical scores [(NIHSS, BI, mRS, Essen) at admission and at discharge]; LCR.

*P < 0.05.

In addition, the IS subtypes, degree of intracranial stenosis, and infarction area were not related to CCR (Supplementary Tables 2B and 3).

## DISCUSSION

### Definition of CR

In the present study, CR, including LCR and CCR in conjunction, represents an accurate measure from a laboratory and clinical perspective. The average life span of the platelet is about 7 days; LCR is a laboratory parameter used for describing the early poor response to clopidogrel within 7 days. The vasodilator-stimulated phosphoprotein (VASP) phosphorylation assay is the most rational evaluation of ADP-induced platelet P2Y12 receptor activation and, as a consequence, a specific marker of the clopidogrel effect [2, 17]. On the other hand, CCR represents the failure of clopidogrel treatment within 6 months, similar to adverse clinical outcomes. In addition, the carriers of *CYP2C19* loss-of-function alleles are considered to be at great risk for IS if treated with clopidogrel, according to our previous studies [2, 4].

### Current clinical status of BPPs in patients with NCIS treated with clopidogrel

Several studies have indicated that BPPs are simple and useful indicators, which can be collected from a plain and routine laboratory test. These BPPs are correlated with the development of adverse events or residual platelet reactivity under antiplatelet therapy in ischemic cardiovascular or cerebrovascular diseases [5–7, 9, 11, 12, 14, 18–20]. However, several studies have indicated that BPPs are not associated with ischemic events [8, 10, 16, 21].

Thrombus formation and platelet activation play a pivotal role in the pathogenesis of acute IS. The estimations of platelet volume and number (which are both determinants of the platelet mass) are consistent with the function and activation of platelets [8]. The MPV is widely used to assess the platelet size and functions, while PC reflects the functions, production, and aging of platelets [14]. Furthermore, P-LCR is the percentage of platelets measuring > 12 fL and is also a routinely assessed marker of platelet size and activity [6, 7, 9, 13]. On the other hand, PDW indicates the morphometric indices of size distribution and is an index of platelet size heterogeneity; a large PDW can be an indicator of the prothrombotic status [22]. Notably, the MPV and PC are the two major indexes reflecting the platelets’ functions and activities [14].

The main finding of the present study was that high baseline MPV (≥ 10.6 fL) and PC (≥ 235 × 10^9^/L) were associated with CCR, exhibiting an inverse relationship with clopidogrel therapy in patients with NCIS: lowest (baseline MPV and PC level) in the non-CCR group and highest (baseline MPV and PC level) in the CCR group. The MPV has been shown to be an independent risk factor of IS in patients with atrial fibrillation [6, 23]; hence, patients with NCIS were selected as the primary research participants in the present study. An elevated MPV level might reflect an increase in reticulated or immature platelets. A high platelet turnover has been shown to be correlated with platelet aggregation and poor response to antiplatelet therapy. Large platelets exert a high metabolic activity and contain more high-density granules, produce more thromboxane B2, express a high level of P-selectin, fibrinogen, and glycoprotein IIb/IIIa receptors, and secrete large amounts of serotonin and β-thromboglobulin. Thus, the larger platelets are more prone to aggregation than the smaller platelets and easily form thrombi [11, 22, 24]. The patients with clinical CR showed a remarkable change in the positive MPV value than that in the clopidogrel responding patients [23, 25]. Reportedly, the level of PC was lower in patients with ischemic cardiovascular diseases than in healthy controls [25]. However, different and conflicting results were presented in other studies [14, 26]. For example, the risk of cardiovascular disease was elevated at PC of 301 × 10^9^/L–450 × 10^9^/L [27]. Therefore, patients with high MPV and PC pre-treatment levels may lead to a predisposition towards the thrombogenic potential [14, 28].

Previous studies have established that the MPV and PC levels are inversely correlated [20]; the PC decreases with increasing MPV. However, the mechanism underlying this inverse relationship is still unclear. One study demonstrated that the stimulations resulting in an increase in PC and platelet volume during thrombopoiesis are regulated by independent mechanisms [22]. Herein, we indicated a positive correlation between MPV and BPRI, whereby a greater MPV was correlated with the expression of surface receptors, indicating an increased readiness of platelets to aggregate [9]. The BPRI reflects the aggregation function of platelets before clopidogrel treatment; a high BPRI might predict a high LCR rate in patients with NCIS [2].

### Predictive risk factors for CCR

In the current study, we found that LCR was related only to *CYP2C19* gene polymorphisms, which might be attributed to the mechanisms of clopidogrel underlying the CYP pathways on the antiplatelet response [3]. Elevated LDL-C levels and DM were correlated with CCR, which indicates that the adverse clinical outcomes of clopidogrel may be affected by VRFs. Moreover, VRFs exhibited a positive correlation with endothelial dysfunction and accelerated the development of atherosclerosis in a synergistic manner. On the other hand, LDL-C promoted atherosclerosis via endothelial damage and increased platelet aggregations [29]. The thromboinflammatory injury may be further aggravated in patients with high MPV, and endothelial cell damage following IS may be more likely to relapse into re-thrombosis in patients with high MPV because of increased thrombogenic potential and antiplatelet agent resistance [28]. The large platelets might promote the thrombotic events in a susceptible patient rather than simply being a consequence of the acute event [11]. With increasing MPV and total PC, the absolute number of aggregated platelets will also increase. Therefore, some BPPs putatively overwhelmed the other factors in determining the response to clopidogrel, as manifested by the elevated MPV and PC, among patients with NCIS, which contain excessive α-granules and release prothrombotic substances; these substances aggravate the inflammation and endothelial dysfunction [7], affecting the future outcomes. We also found that the large number of female patients in the CCR, elevated MPV, and PC groups was not only because of the phenomenon that women with specific characteristics are associated with the occurrence of stroke [30] but also because it is unclear whether changes in endogenous estrogen levels can lead to altered platelet function. In addition, the high HbA1c level might be related to LCR and increased MPV and PC; the increased levels of HbA1c positively correlated with increased platelet aggregation, turnover, and activation. We found that the NIHSS score at discharge was also a risk predictor for CCR, whereby a higher NIHSS score indicated a more severe neurological deficit. We also found that patients with LCR were more likely to develop CCR, which is in line with our previous findings [2].

### The internal interaction of BPPs and non-genetic risk factors

The PDW, PCT, and P-LCR did not correlate strongly with LCR and did not change in the CCR cases. Thus, a large variation in platelet size indicates a shift towards the population of bigger platelets.

In addition, MPV and PC were both found not to be discriminative regarding LCR or CCR, according to the ROC curve analysis. We also verified if there was a correlation between NLR and MPV/PC, but no conclusion could be drawn. However, the NLR was significantly higher in patients with CCR than in the non-CCR group. One reason is that the role of BPPs in the prognosis of NCIS has not been elucidated. Atherosclerosis is a chronic and complex progressive inflammatory disease and not merely the passive accumulation of lipids within the arterial walls. Moreover, thrombogenesis and inflammation interact and reinforce each other in the pathogenesis of IS [8]. The platelet size is regulated by various intrinsic and extrinsic elements [8, 25]. Thus, the relationship between platelets and CCR might be a result of the complex influence of VRFs, and the inflammatory and therapeutic sensitivity. All these risk factors might conjointly contribute, at least partially, to the increased MPV and PC values in patients with NCIS with clopidogrel therapy. Moreover, higher MPV and PC values could also impact the adverse clinical outcomes conversely through these risk factors. Another potential explanation is that different methods were used to measure BPPs in different laboratories. The negative result may be partially explained by the insufficient exclusion criteria and different grouping methods.

A “one size fits all” approach to the antiplatelet treatment of NCIS is probably flawed. Targeted therapeutic changes to ease specifically the customization of antiplatelet therapy from the comprehensive perspective of BPPs, platelet functional tests, *CYP2C19* gene variants, and other more non-genetic risk factors would likely provide a reasonable solution.

Nevertheless, the limitations of the current study should be mentioned. First, a potential bias due to the relatively small sample size and because no other gene polymorphisms were investigated cannot be excluded. Second, the PRI in the 6th month and the interaction of each BPP and VRF has not been evaluated. Third, the active metabolites of clopidogrel in various genotypes, as well as the plasma clopidogrel levels in NCIS, were not detected. Fourth, the recurrence rate of ischemic events may not be substantial owing to the short follow-up period. Fifth, the CCR was defined for all statistical analysis irrespective of the time variable. Therefore, additional multi-center clinical trials with larger samples are warranted to confirm our findings.

## MATERIALS AND METHODS

### Ethical considerations

The study was approved by the Medical Ethics Committee of the First Affiliated Hospital of Jinan University, China, and registered in the World Health Organization Clinical Trial Registry (registration number: ChiCTR-ONC-13003406). Written informed consent was obtained from each participant before enrollment.

### Study population

Between February, 2012 and February, 2016, data of all consecutive patients within 3 days of acute NCIS onset receiving clopidogrel (Plavix^®^) were prospectively collected and retrospectively analyzed at the First Affiliated Hospital of Jinan University, Department of Neurology. The clinical data were assimilated from hospital records by the attending physicians. After discharge, the clinical parameters were collected for 6 months by hospital visit or telephone. The scheduled telephonic follow-up was performed every month to address any queries, encourage compliance, and document any side-effects. The researchers evaluating the clinical events were blinded to the other data, such as genotypes. The patients with NCIS were treated and managed according to the relevant clinical guidelines [1]. Blood samples (4 mL) were obtained from the antecubital vein before and 7 days after clopidogrel (Plavix^®^) treatment for BPPs, gene polymorphisms, and early platelet response. A schematic of the current study is shown in Supplementary Figure 1.

### Baseline platelet indicators

All participants underwent routine blood tests for the measurements of BPPs (MPV, PC, PDW, PCT, and P-LCR) before antiplatelet therapy in the case of emergency or during admission. Blood samples were withdrawn with careful venipuncture from the antecubital vein, collected into ethylenediaminetetraacetic acid (EDTA) tubes, and preserved at room temperature. In this study, the BPPs were estimated within 2 hours of samplings. All measurements were processed on the XE-5000 System (Sysmex Corporation, Japan).

The reference limits of each BPP were as follows: MPV [8.0–2.5 fL]; PC [100–300 × 10^9^/L]; PDW [9.0–17.0 fl]; PCT [0.16–0.40%]; and P-LCR [13–43%].

### Definition of non-genetic risk factors

The gathered non-genetic risk data included the patients’ risk demographic characteristics, VRFs [31], clinical scores, administered drugs, and available laboratory data.

#### The VRFs

Hypertension was defined as a self-reported history or use of antihypertensive medication or a diagnosis of hypertension at discharge. DM was defined as a self-reported history or use of insulin or oral hypoglycemic treatment, glycosylated hemoglobin level ≥ 7%, or a diagnosis of DM at discharge. Hyperlipidemia was defined as a self-reported history of use of lipid-lowering therapy or current treatment with lipid-lowering therapy, an LDL-C level of 2.6 mmol/L at admission, or a diagnosis of hyperlipidemia at discharge. A history of IS or transient ischemic attack was also defined. Coronary artery disease was defined as a reported history of myocardial infarction, angina pectoris, positive stress test, or cardiac surgery/intervention. A current smoker was defined as a patient who smoked ≥1 cigarette per day continuously for 6 months. A heavy drinker was defined as a patient who drank >2 units of alcohol per day on average (for male patients) or >1 unit of alcohol per day on average (for female patients).

#### The clinical scores

The neurological function of each patient was assessed using the NIHSS, Barthel Index (BI), Modified Rankin Scale (mRS), and Essen scores by the attending neurologists at admission and at discharge.

#### The IS subtypes

The patients with NCIS were classified into large-artery atherothrombosis (LAA) and cerebral small-vessel disease (cSVD) subtypes, according to the Chinese Ischemic Stroke Subclassification (CISS) criteria [32].

The infarction areas were categorized as anterior circulation, posterior circulation, and both. The degree of intracranial stenosis on magnetic resonance angiography or digital subtraction angiography was determined by the method of the WASID study [31], and categorized as mild, moderate, or severe.

### Definition of clopidogrel resistance

#### The LCR

**Figure 3:**
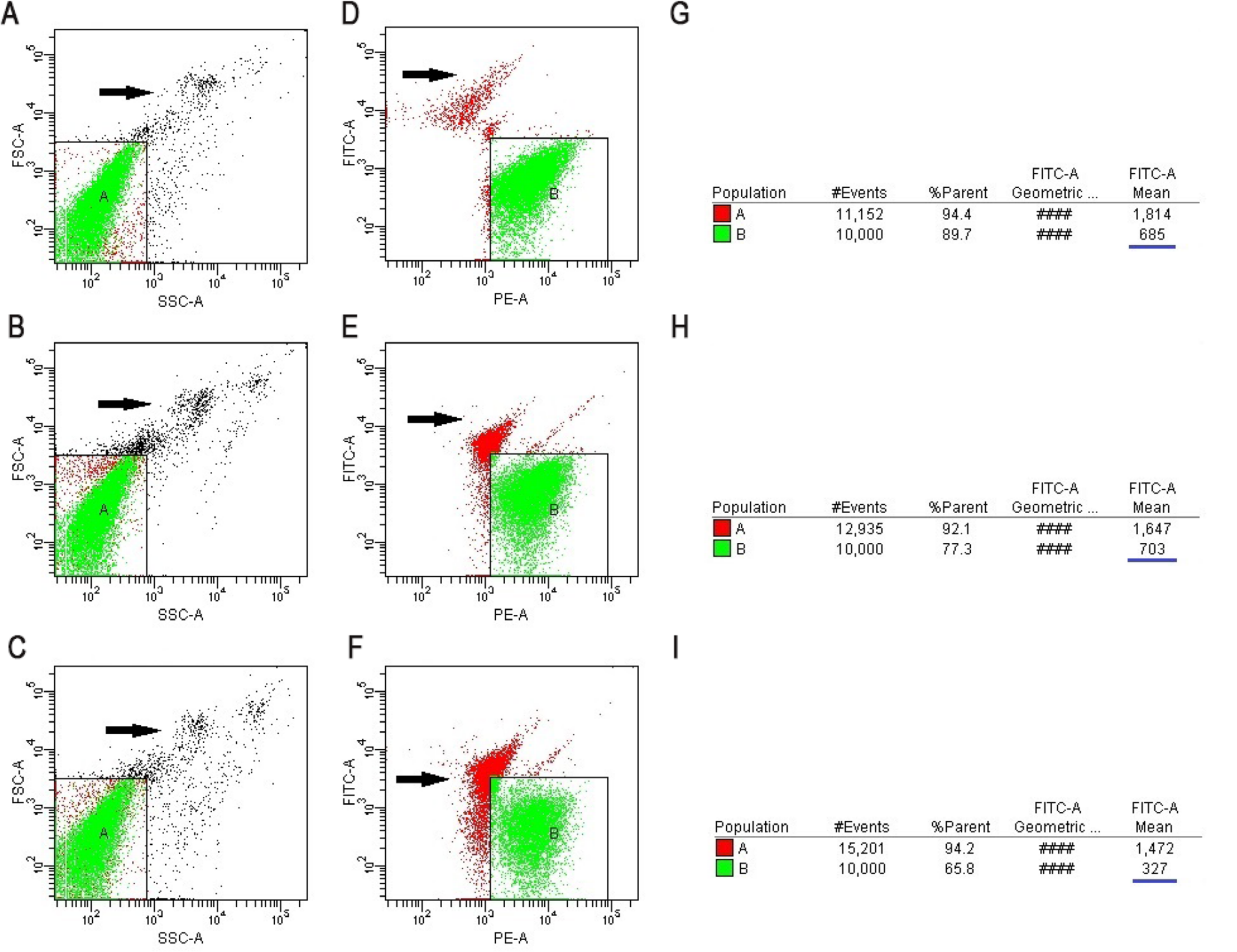
The detection procedure of PRI using flow cytometry The T1, T2 and T3 tubes have the same position in regions A and B. The T1 and T2 tubes were experimental tubes and the T3 tubes were controls. (**A**–**C**) Setting region A of the T1, T2 and T3 tubes (white blood cells should be excluded from the region; black arrow indicates white blood cells), respectively. (**D**) Setting region B of the T1 tube (black arrow indicates the cell debris): a group of platelets that are activated by PGE1 and are shown in the combination of anti-VASP phosphorylated monoclonal antibodies. (**E**) Setting region B of the T2 tube (black arrow indicates the cell debris): the antibody combining with the granule group of the combination of ADP and P2Y12 and then activated by PGE1. (**F**) Setting region B of the T3 tube (black arrow indicates the cell debris): the negative control granule group of negative VASP phosphorylated antibody and PGE1/ADP combined with platelet. (**G**–**I**) Analysis of MFI of the T1, T2, and T3 tubes using CELL Quest software (The blue underline).

Flow cytometric measurements of the level of VASP phosphorylation was used for assessing the early platelet response to clopidogrel therapy [2]. The detection procedure of PRI using flow cytometry is shown in Figure 3. The PRI included the values at BPRI before clopidogrel treatment and at PPRI for 7 days. After obtaining the BPRI values, patients were administered a 300-mg oral loading dose of clopidogrel followed by a standard dosage of 75 mg/day for at least 6 months. The PPRI values were obtained after 7 days. The assays were conducted by using a Platelet VASP-FCM kit (Biocytex, Marseille, France) according to the manufacturer’s instructions (Analysis and Testing Center, Jinan University).

The LCR was defined as PPRI > 50% [2, 33], which represented early poor response to clopidogrel.

#### The CCR

The CCR is the equivalent to adverse clinical outcomes, which were defined as the onset of progressive IS (an increase of NIHSS score ≥ 2) during admission, recurrence of IS [34], or occurrence of other ischemic diseases within 6 months [2]. These events were confirmed by reviewing the hospital discharge reports. Alternatively, the data were collected by telephonic follow-up.

The IS recurrence was verified at the index hospitals based on the presence of new neurological deficits documented in the medical records combined with CT or MRI images [34]. At 3 and 6 months after discharge, the patients or their relatives were contacted over the telephone by trained research personnel at the First Affiliated Hospital of Jinan University and asked whether the patients had developed new symptoms or had been hospitalized again with another stroke. An experienced neurologist reviewed the patients’ medical records to ensure a reliable diagnosis of IS recurrence. In patients with an unclear event who were not hospitalized, they would be adjudicated by a stroke neurologist and the principle investigator [35].

### Genetic analysis

The polymorphisms of *CYP2C19* gene loci within two genotypes, including *CYP2C19* (636G>A) and *CYP2C19* (681G>A), were screened. The chain termination method was used to sequence the above-mentioned genotypes (ABI 3730, Applied Biosystems Inc., Foster City, CA) [2], which were identified by the BGI Company (Beijing, China).

### Statistical analysis

All statistical analyses were performed using SPSS version 19.0A (IBM, Armonk, NY, USA). The measurement data were represented as the mean ± standard deviation. The categorical data were presented as percentage. Groups were compared by independent-samples *t*-test. Pearson χ^2^ test and Fisher’s exact test were used for assessing the categorical variables as appropriate. The Spearman’s correlation coefficient was computed to examine the association between the two continuous variables. A *P*-value < 0.05 was considered statistically significant. The ROC curve analysis determined the sensitivity and specificity with 95% CI for each BPP at the set cut-off values. At the set cut-off value, sensitivity and specificity over 60% and AUC over 0.7 were considered statistically significant.

Significant predictive risk factors for LCR and CCR were identified by non-conditional logistic regression analysis. The ORs and 95% CIs were calculated for each variable. The factors with a *P*-value < 0.10 from the univariate analysis were entered into the model. Variables with OR > 1 and *P* < 0.05 in the univariate analysis were included in the binary regression model as positive prediction risk factors.

## SUPPLEMENTARY MATERIALS FIGURES AND TABLES



## Abbreviations

IS: ischemic stroke
NCIS: non-cardioembolic ischemic stroke
CR: clopidogrel resistance
BPPs: baseline platelet parameters
MPV: mean platelet volume
P-LCR: platelet-large cell ratio
PDW: platelet distribution width
PC: platelet count
PCT: plateletocrit
LCR: laboratory clopidogrel resistance
CCR: clinical clopidogrel resistance
BPRI: baseline platelet reactivity index
PPRI: post-treatment platelet reactivity index
HbA1c: haemoglobin A1c
NLR: neutrophil to lymphocyte ratio
ROC analysis: Receiver operating characteristic analysis
AUC: area under the curve
VRFs: vascular risk factors
DM: diabetes mellitus
LDL-C: low-density lipoprotein cholesterol
NIHSS: National Institutes of Health Stroke Scale
VASP: vasodilator-stimulated phosphoprotein
EDTA: ethylenediaminetetraacetic acid
BI: Barthel Index
mRS: modified Rankin Scale
LAA: large-artery atherothrombosis
cSVD: cerebral small-vessel disease
PRI: platelet reactivity index
CI: confidence interval
ORs: odds ratios
CHD: coronary heart disease
TIA: transient ischemic attack
HDL: high-density lipoprotein
