# Supplementary material for: Baseline platelet parameters for predicting early platelet response and clinical outcomes in patients with non-cardioembolic ischemic stroke treated with clopidogrel
Source: Oncotarget. 2017 Oct 7;8(55):93771–84. doi: 10.18632/oncotarget.21622 (PMC5706834; doi:10.18632/oncotarget.21622)
Supplement: Supplementary file 1 [file oncotarget-08-93771-s001.pdf]

Baseline platelet parameters for predicting early platelet response and clinical outcomes in patients with non-cardioembolic ischemic stroke treated with clopidogrel

SUPPLEMENTARY MATERIALS

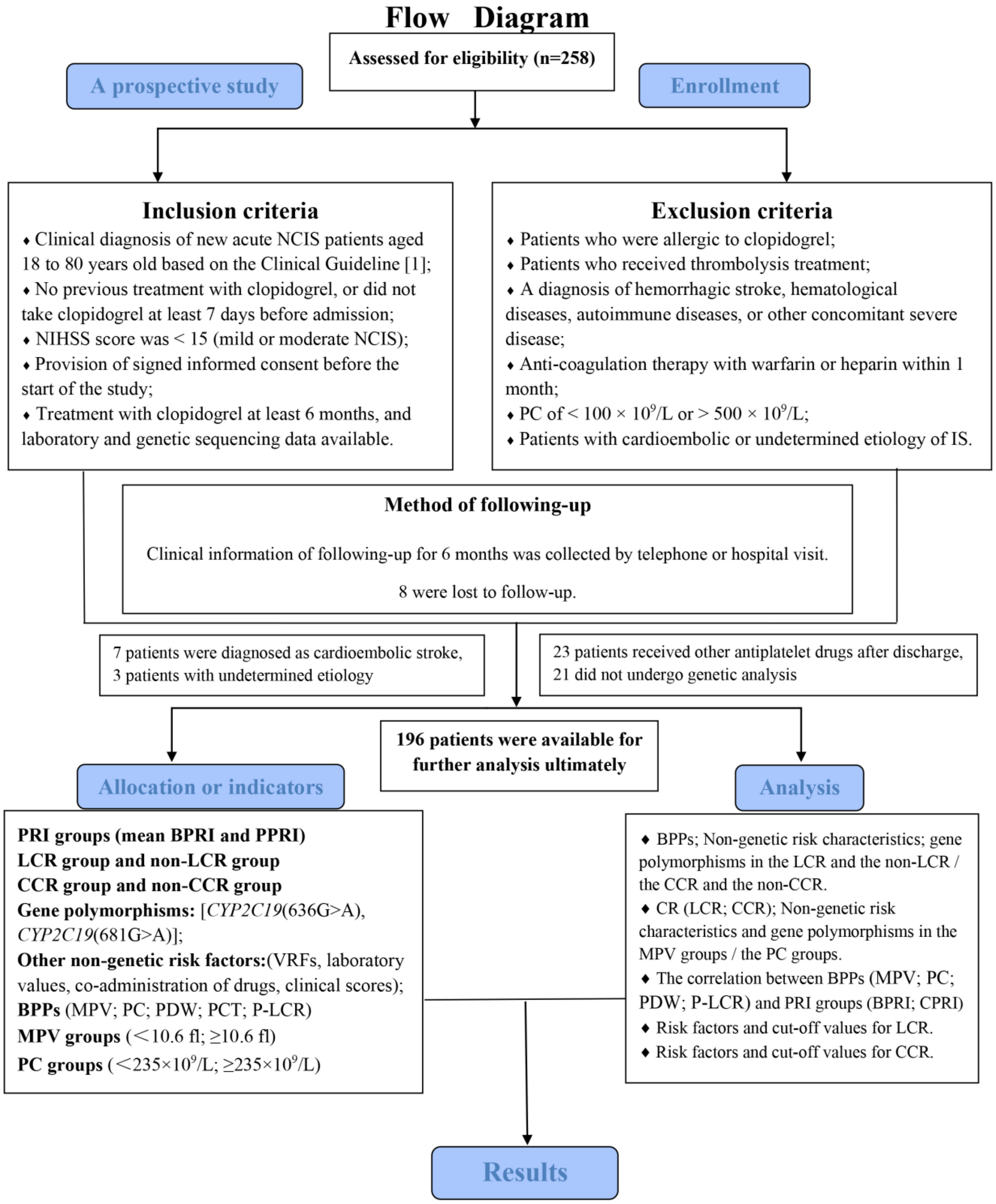

Supplementary Figure 1: Flow diagram of the study. NCIS, non-cardioembolic ischemic stroke; NIHSS, National Institutes of Health Stroke Scale; PC, platelet count; IS, ischemic stroke; PRI, platelet reactivity index; BPRI, baseline of platelet reactivity index; PPRI, post-treatment of clopidogrel for 7 days; LCR, laboratory clopidogrel resistance; CCR, clinical clopidogrel resistance; VRFs, vascular risk factors; BPPs, baseline platelet parameters; MPV, mean platelet volume; PDW, platelet distribution width; PCT, plateletocrit; P-LCR, platelet-large cell ratio

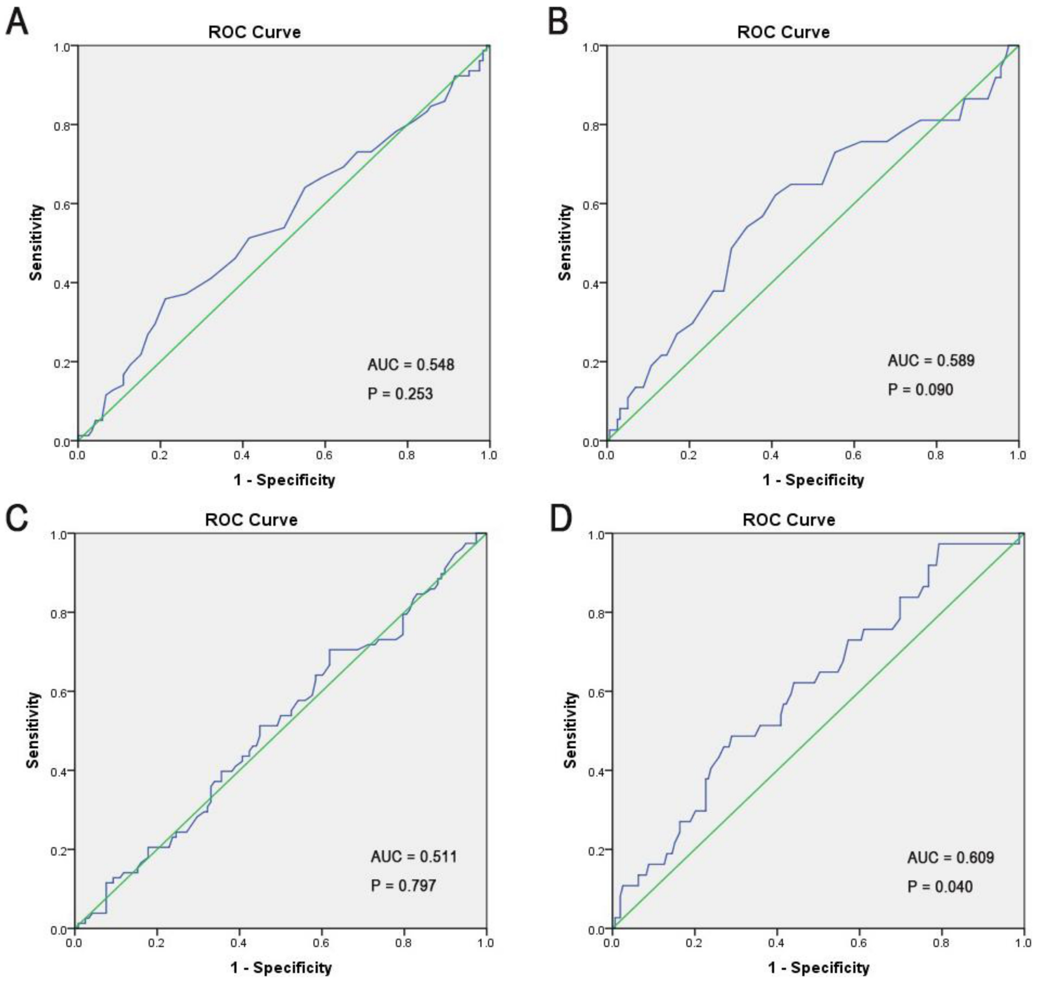

Supplementary Figure 2: ROC curve analysis of MPV/PC in prediction of CR. (**A**) ROC curve analysis of MPV in prediction of LCR. (**B**) ROC curve analysis of MPV in prediction of CCR. (**C**) ROC curve analysis of PC in prediction of LCR. (**D**) ROC curve analysis of PC in prediction of CCR.

Supplementary Table 1A: Genetic characteristics of the MPV groups

Gene polymorphisms	MPV<10.6 fL (*n* = 95)	MPV≥10.6 fL (*n* = 101)	*P* value
CYP2C19 (636G>A)			0.927
GG	70 (73.68)	75 (74.26)	
GA/AA	25 (26.32)	26 (25.74)	
CYP2C19 (681G>A)			0.715
GG	57 (60.00)	58 (57.43)	
GA/AA	38 (40.00)	43 (42.57)	

Supplementary Table 1B: Genetic characteristics of the PC groups

Characteristics	PC<235×109/L (*n* = 110)	PC≥235×109/L (*n* = 86)	*P* value
CYP2C19 (636G>A)			0.595
GG	83 (75.45)	62 (72.09)	
GA/AA	27 (24.55)	24 (27.91)	
CYP2C19 (681G>A)			0.874
GG	64 (58.18)	51 (59.30)	
GA/AA	46 (41.82)	35 (40.70)	

Abbreviations as in Table 1.
Categorical data are expressed as *n* (%).

Supplementary Table 2A: IS subtypes in LCR VS. Non-LCR

IS subtypes	LCR (*n* = 78)	Non-LCR (*n* = 118)	*P*
			0.678
LAA	57 (73.08)	83 (70.34)	
cSVD	21 (26.92)	35 (29.66)	

Supplementary Table 2B: IS subtypes in CCR VS. Non-CCR

IS subtypes	CCR (*n* = 37)	Non-CCR (*n* = 159)	*P*
			0.065
LAA, *n* (%)	31 (83.78)	109 (68.55)	
cSVD, *n* (%)	6 (16.22)	50 (31.45)	

LAA, large-artery atherothrombosis; cSVD, cerebral small-vessel disease.
Other abbreviations as in Table 1.
Categorical data are expressed as *n* (%).

Supplementary Table 3: Infarction area and degree of stenosis in CCR VS. Non-CCR

	CCR (*n* = 37)	Non-CCR (*n* = 159)	*P*
Infarction area			
Anterior circulation	18 (48.65)	75 (47.17)	0.871
Posterior circulation	12 (32.43)	51 (32.08)	0.967
Both anterior and posterior circulation	7 (18.92)	33 (20.75)	0.803
Degree of stenosis			
Mild degree	9 (24.32)	61 (38.37)	0.108
Moderate degree	15 (40.54)	63 (39.62)	0.918
Severe degree	13 (35.14)	35 (22.01)	0.095

Abbreviations as in Table 1.
Categorical data are expressed as *n* (%).
